# Investigation of the Enhancement Effect of *Evodia rutaecarpa* Volatile Oil on Transdermal Delivery of Total Glucosides of Paeony: Mechanistic Insight Based on Interactions Among Drug, Enhancer, and Skin

**DOI:** 10.3390/pharmaceutics18040433

**Published:** 2026-03-31

**Authors:** Zhanghong Yao, Fei Song, Yan Liang, Yunfeng Liu, Weifeng Zhu, Yongmei Guan, Lili Liu

**Affiliations:** 1 College of Pharmacy, Jiangxi University of Chinese Medicine, Nanchang 330004, China; yzh20001101@126.com (Z.Y.); sf20031005@163.com (F.S.); 2 Key Laboratory of Modern Preparation of TCM, Ministry of Education, Jiangxi University of Chinese Medicine, Nanchang 330004, China; liangyan202302@163.com (Y.L.); lyf737422@163.com (Y.L.); zwf0322@126.com (W.Z.)

**Keywords:** *Evodia rutaecarpa* volatile oil, total glucosides of peony, transdermal permeation enhancer, interaction mechanism

## Abstract

**Background**: Total glycosides of peony (TGP) have therapeutic potential for immune-related and inflammatory skin diseases, but their skin absorption is not satisfactory. This study aims to investigate how *Evodia rutaecarpa* volatile oil (VO-ER) enhances the permeability of TGP. **Methods**: Safety assessment was conducted through cell delivery and skin erythema tests. The chemical composition of VO-ER was identified via GC-MS. The study was conducted using modified Franz diffusion cells, microdialysis, confocal laser scanning microscopy (CLSM), attenuated total reflection–Fourier transform infrared spectroscopy (ATR-FTIR), molecular docking and molecular dynamics simulations (MD), laser Doppler flowmetry (LDF), and the western blotting method. **Results**: The study found that VO-ER promotes the permeation of total glycosides of peony in a concentration-dependent manner by disrupting the intercellular lipid tissue structure, downregulating the expression of claudin-1, claudin-7, and occludin, and improving local microcirculation, thereby promoting the absorption of TGP. **Conclusions**: VO-ER enhances the transdermal absorption of TGP through multiple mechanisms, such as disrupting the skin lipid barrier, downregulating tight junction proteins, and improving local skin microcirculation. This study provides a theoretical basis for VO-ER as a safe and effective new transdermal penetration enhancer, offering support for the development of topical preparations containing *Evodia rutaecarpa* and *Paeonia lactiflora*.

## 1. Introduction

*Evodia rutaecarpa* is a traditional Chinese herbal medicine that has been extensively used topically throughout history, frequently employed for treating stubborn conditions externally and serving as a key substance in acupoint application therapies [1]. The Divine Farmer’s Materia Medica records the following property: “...opening couli, possessing the function of opening the skin.” *Evodia rutaecarpa* and *Paeonia lactiflora* are often used in combination. In the external treatment monograph “Li Yue Pian Wen,” there are 32 prescriptions involving the combination of the two. *Evodia rutaecarpa* volatile oil (VO-ER) is one of the active components of *Evodia rutaecarpa*. Total glucosides of peony (TGP) is an effective component extracted from *Paeonia lactiflora* that has significant therapeutic effects in the treatment of various chronic diseases and autoimmune diseases. Its clinical application in immune-related and inflammatory skin diseases has been increasing year by year [2]. Previous literature has shown that VO-ER can significantly promote the transdermal absorption of paeoniflorin (PF) in TGP [3], but its penetration-enhancing patterns and mechanisms remain unclear.

The skin barrier is an important component of the skin. The “epidermal wall theory” compares the skin to a “mortar” structure, with corneocytes as bricks, intercellular lipids as mortar, and tight junction proteins as steel bars. These three components tightly connect the epidermis, forming the skin barrier.

It is reported that the penetration-enhancing mechanisms of volatile oils in traditional Chinese medicine are frequently associated with their ability to enhance skin lipid fluidity and dissolve/extract intercellular lipids [4]. Therefore, it is possible that VO-ER can disrupt the ordered arrangement of lipids in the stratum corneum, extract lipid components, or fluidize lipids to improve skin permeability, thereby reducing skin transport resistance and regulating the transdermal absorption of TGP.

Corneocytes, as the “bricks” in the epidermal wall theory, and tight junction proteins in the skin, as the “steel bars,” play important roles in maintaining the skin’s barrier function, regulating substance transport, maintaining cell polarity, and participating in skin repair and the organizational structure of keratin in the stratum corneum, thereby enhancing skin permeability [5]. Phenolic compounds in clove oil—such as eugenol, isoeugenol, and caryophyllene—denature proteins, including keratin, in the stratum corneum, which subsequently alters the permeability of phospholipids in cell membranes [6]. Whether VO-ER enhances the transdermal transport of PF from TGP by acting on keratinocytes and tight junction proteins—thereby facilitating the “opening of the couli”—warrants further investigation.

*Evodia rutaecarpa* is pungent with a hot nature. Modern pharmacological studies have shown that *Evodia rutaecarpa* extracts can dilate blood vessels and increase blood flow. Our previous studies found that increasing skin blood flow can increase the elimination rate of drugs in the skin, resulting in a sink effect and promoting drug transdermal absorption [7]. Therefore, we speculated that VO-ER may regulate the transdermal transport and absorption of TGP by altering skin microcirculation.

This study elucidates the mechanistic basis of VO-ER in facilitating the transdermal transport of TGP through skin barrier modulation—scientifically validating its classical “opening the interstitial space between the skin and muscles (termed couli in traditional Chinese medicine)“ function. Our findings offer novel mechanistic insights into the penetration-enhancing properties of volatile oils, while facilitating the development of topical formulations containing *Evodia rutaecarpa* and *Paeonia lactiflora*.

## 2. Materials and Methods

### 2.1. Animals

Male Sprague–Dawley (SD) rats (6–8 weeks old, weighing 180–220 g, purchased from the Experimental Animal Technology Center of Jiangxi University of Chinese Medicine) were housed in a standard laboratory animal environment [temperature: (22 ± 2) °C; relative humidity: (55 ± 5)%; light/dark cycle: 12 h/12 h] with free access to food and water. All SD rats were acclimated for one week before the experiment to adapt to the new environment. All rats were anesthetized with urethane (20% *w*/*v*, i.p.) before euthanasia to minimize discomfort. All experimental protocols were approved by the Animal Ethics and Welfare Committee of Jiangxi University of Chinese Medicine (Certificate number: JZLLSC20250585, approval date: 10 October 2025).

### 2.2. Reagents

*Evodia rutaecarpa* and *Paeonia lactiflora* were purchased from Jiangxi Jiangzhong Chinese Herbal Decoction Pieces Co., Ltd. (Jiujiang, China). Paeoniflorin and paeonolide were purchased from the National Institute for Food and Drug Control (Beijing, China). HPLC-grade methanol and acetonitrile were purchased from Hubei Ferton Science and Technology Co., Ltd. (Wuhan, China). Phosphoric acid and acetic acid were purchased from Shanghai Aladdin Bio-Chem Technology Co., Ltd. (Shanghai, China). Anhydrous sodium sulfate and ethyl acetate were purchased from Xilong Scientific Co., Ltd. (Guangzhou, China).

### 2.3. Preparation of Evodia rutaecarpa Volatile Oil

VO-ER was prepared through steam distillation following the method of Yang Y.Q. et al., 2023 [8], and its main components were determined via GC-MS (Agilent 7890A/5975C, Agilent Technologies, Santa Clara, CA, USA).

Separation was performed on a fused-silica capillary column (30 m × 0.25 mm i.d., 0.25 μm film thickness). High-purity helium (99.999%) was used as the carrier gas at a constant flow rate of 1.0 mL/min. The injector temperature was set at 250 °C. The column temperature program was as follows: the initial temperature was held at 70 °C for 3 min, then increased to 130 °C at a rate of 5 °C/min, subsequently raised to 160 °C at 2 °C/min, and finally increased to 260 °C at 10 °C/min and held for 5 min. The injection volume was 1.0 μL, with a split ratio of 20:1.

The mass spectrometer was operated in electron impact (EI) mode at 70 eV. The ion source temperature was maintained at 230 °C, the quadrupole temperature was set at 150 °C, and the interface temperature was set at 280 °C. Mass spectra were acquired in full-scan mode over a mass range of m/z 30–500, and compound identification was performed by matching the acquired spectra with the NIST 11 mass spectral library.

### 2.4. Biosafety Assessment

To evaluate the biosafety of VO-ER and its combinations, cytotoxicity and skin erythema tests were performed. The cytotoxicity of VO-ER and TGP was assessed using HaCaT cells via the MTT assay, while skin irritation was evaluated in rats by observing erythema following topical application.

#### 2.4.1. Cytotoxicity Evaluation

Human immortalized keratinocytes (HaCaT cells) were purchased from the Cell Bank of the Chinese Academy of Sciences. HaCaT cells were seeded in 96-well plates at a density of 7 × 10^3^ cells per well and cultured for 24 h. Subsequently, different concentrations of VO-ER (0.02~0.60 mg/mL) and TGP solutions containing different concentrations of VO-ER were added to the cells and cultured for 24 h. Then, 100 μL of 10% MTT reagent was added to each well, and the plates were incubated at 37 °C for 6 h. The culture medium was removed, and 110 μL of Formazan dissolution solution was added. The plates were shaken at 100 rpm for 10 min to dissolve the Formazan. Absorbance was measured at 490 nm using a multi-functional microplate reader (S/N502000011, Tecan Trading AG, Shanghai, China). Cell viability was calculated as a percentage relative to untreated control cells.

#### 2.4.2. Skin Erythema Analysis

The skin irritation of rats was evaluated via in vivo skin erythema analysis. The skin erythema index (EI) was measured using a skin multifunctional elasticity tester (MPA580, CK, Cologne, Germany). Solutions containing 1%, 3%, and 5% (*w*/*w*) VO-ER and 1%, 3%, and 5% (*w*/*w*) VO-ER solutions containing 180 mg/mL TGP were prepared as the administration groups. The hair on both sides of the rat’s back was shaved, with a size range of 1.5 cm × 1.5 cm. Next, 0.5 mL of pure water was applied to the right side of the skin as the blank group, and 0.5 mL of the corresponding administration solution was applied to the left side. The skin erythema index of each group was measured before administration, 2 h after administration, and 4 h after administration. The results were expressed as the change value of the skin erythema index (skin erythema index after administration/skin erythema index before administration).

### 2.5. Ex Vivo Skin Permeation Study

#### 2.5.1. Quantitative Analysis of Paeoniflorin

Quantitative analysis was conducted using high-performance liquid chromatography (HPLC) (Agilent 1260, Agilent Technologies, Santa Clara, CA, USA) with a Cosmosil C18-MS-II HPLC column (250 mm × 4.6 mm, 5 μm, Cosmosil, Beijing, China). The mobile phase was acetonitrile and 0.1% phosphoric acid solution (21:79, *v*/*v*), with a flow rate of 1 mL·min^−1^ and a detection wavelength of 230 nm.

#### 2.5.2. Preparation of Rat Skin

According to the study by Myburgh J. et al., 2023 [9], rats were anesthetized with urethane (20% *w*/*v*, i.p.). The abdominal hair was removed first with an electric shaver and then the skin was carefully shaved with a razor. Full-thickness skin was excised from the shaved abdominal area. After shaving, the rats were euthanized, and the abdominal skin was removed. Subcutaneous fat was removed using surgical scissors and forceps. The integrity of the skin was carefully checked to ensure that it was undamaged. The skin samples were stored at −80 °C and used within one month. Before the experiment, the skin was slowly thawed to room temperature.

#### 2.5.3. Ex Vivo Skin Permeation Assay Using Franz Diffusion Cells

A vertical diffusion cell (KX-V/HDP, Dalian Kexiang Intelligent Transdermal Diffusion Instrument, Dalian, China) was used, with an effective contact area of 1.77 cm^2^ between the skin and the receptor chamber and a receptor chamber volume of 10.0 mL. Test solutions were prepared by dissolving 180 mg of TGP in 1.0 mL of ultrapure water and adding 1%, 3%, and 5% (*w*/*w*) VO-ER, respectively. The solution without VO-ER was used as the control group. The treated rat skin was placed between the donor and receptor chambers. The receptor solution was physiological saline. Next, 1 mL of the test solution was added to the donor chamber, and 10 mL of the receptor solution was added to the receptor chamber. A 1.0 mL aliquot of the receptor solution was collected at 1, 2, 4, 6, 8, 10, 12, and 24 h after administration, and the same volume of physiological saline was added. The analysis method is described in Section 2.5.1. The concentration of paeoniflorin in the receptor solution was determined, and the cumulative skin permeation amount per unit area (Q24h) was calculated. A linear regression of Q_n_ against time t was conducted to draw the cumulative drug permeation curve. The slope of the curve was the drug permeation rate, Jss (μg·cm^−2^·h^−1^).

### 2.6. In Vivo Skin Permeation Study

#### 2.6.1. LC-MS/MS

The LC-MS/MS system (AB TRIPLE QUDA 4500, AB SCIEX, Singapore) was used for quantitative analysis of PF in the in vivo absorption samples. The chromatographic conditions included a Cosmosil C18-MS-II column (150 mm × 2.0 mm, 5 μm, Cosmosil, Beijing, China). The mobile phase consisted of solvent A (0.1% formic acid solution) and B (methanol), with a ratio of A to B of 32:68. The flow rate was 0.3 mL·min^−1^, and the injection volume was 2 μL. The LC-MS/MS system was operated in negative ion mode with MRM, with an ion spray voltage of 2000 V, ion transfer tube temperature of 280 °C, and vaporization temperature of 282 °C. The ion transitions (m/z) for the quantitative analysis of PF and geniposide were 525.1/449.2, 433.2/225.1, and 387.2/123.1, respectively. The method for determining PF was validated, with a retention time of 2.07 min. The calibration curve was linear within the range of 1~500 ng mL^−1^ (R^2^ = 0.9997). The intra-day precision was 3.81%, the inter-day precision was 5.07%, and the freeze–thaw stability was 4.67%.

#### 2.6.2. In Vivo Microdialysis Experiment

Sixteen rats weighing 180~220 g were randomly divided into 4 groups. One group was only administered 0.5 mL of 180 mg mL^−1^ TGP solution, while the other three groups were administered 0.5 mL of 180 mg mL^−1^ TGP solution containing different concentrations of VO-ER (1%, 3%, and 5%, *w*/*w*). Before administration, the abdominal hair of the rats was shaved and washed with physiological saline. The drug solutions were carried by cotton, which was fixed on the abdominal area of the rats after shaving the hair 12 h in advance. The probe was implanted subcutaneously in the hairless area, and physiological saline was used as the perfusion fluid. A microinjection pump was used to perfuse at a flow rate of 1 μL·min^−1^. Samples were collected every 0.5 h for a total of 10 h, resulting in 20 samples. After collection, 30 μL of geniposide solution was added to each sample, mixed via vortexing, and stored at −80 °C. The concentration of PF was determined according to the method described in Section 2.6.1.

### 2.7. Attenuated Total Reflection–Fourier Transform Infrared Spectroscopy (ATR-FTIR)

The interaction between VO-ER and skin lipids was studied using ATR-FTIR (Spectrum, Perkin, MA, USA) [10]. The skin was washed with physiological saline 24 h after administration of the test solution, and the treated area was removed and dried. The skin samples were then placed in a vacuum-drying oven at 37 °C for drying, and the resulting samples were used for analysis. The skin samples were placed on a ZnSe ATR crystal, with a reflection prism (ZnSe) incident angle of 45°. Scans were performed 64 times with a resolution of 4 cm^−1^ and a scan range of 4000–400 cm^−1^. The spectra were processed using OPUS 10.4.2.

### 2.8. Molecular Docking

The main components of VO-ER, such as β-pinene, γ-terpinene, and (Z)-β-ocimene, were retrieved from the PubChem database as ligands. The main lipid components [11], including ceramide 1, ceramide 3, ceramide 6, ceramide 7, cholesterol, tetracosanoic acid, and hexacosanoic acid, were retrieved as receptors. In addition, three keratins closely associated with the skin’s barrier function, namely keratin 1 (KRT1), keratin 10 (KRT10), and keratin 14 (KRT14), were selected as receptors. These ligands and receptors were processed and formatted using Chem 3D 22.0 software and then imported into AutoDock-Tools 1.5.6 software. The relevant parameters of the software were adjusted, and docking was performed. The docking results were imported into PyMOL 2.5.5 software for visualization and drawing. The docking energy (Ea) between each receptor and ligand forming hydrogen bonds or conjugated bonds was calculated.

### 2.9. Molecular Dynamics Simulation (MD)

The structures of β-pinene, γ-terpinene, (Z)-β-ocimene, and PF were generated and geometrically optimized. The POPC lipid bilayer was constructed using the CHARMM-GUI lipid builder, with 64 lipid molecules placed in each layer to form a bilayer containing 128 lipids. The hydration level of the POPC lipid solution included 37 water molecules. All MD simulations were conducted on a Linux system using the Amber simulation package (AmberTools, version 14.0) under the lipid14 force field. After equilibrating for 100 ns in the isothermal-isobaric ensemble, β-pinene, γ-terpinene, and (Z)-β-ocimene were simulated in the POPC lipid bilayer for 50 ns. The 50 ns dynamics simulation process was generated by calculating the mean square displacement (MSD) curve with respect to the simulation time, and the movement trajectories of β-pinene, γ-terpinene, (Z)-β-ocimene, and PF in the lipid bilayer were obtained before and after combination [12,13].

### 2.10. Confocal Laser Scanning Microscopy (CLSM)

Referring to the research methods of Yang N. et al., 2024 [14] and Ruan et al., 2023 [10], sodium fluorescein and Nile Red were selected as fluorescent probes. The experimental steps were the same as those in Section 2.5, except that sodium fluorescein and Nile Red were used instead of PF. The control group was transdermally administered for 4 h, and each experimental group was transdermally administered for 1, 2, 4, 6, 8, and 12 h. The skin of each group was fixed in a universal tissue fixative, and paraffin-embedded tissue sections were prepared to obtain skin section samples containing fluorescent substances. The samples were imaged using a laser confocal microscope (Leica TCS SP5, Leica Microsystems, Wetzlar, Germany). The excitation wavelength for sodium fluorescein was 488 nm, and that for Nile Red was 514 nm.

### 2.11. Western Blot

After drug administration to the HaCaT cells, they were washed with PBS, scraped into a sterile enzyme-free centrifuge tube, and lysed with RIPA lysis buffer and PMSF on ice. The lysate was centrifuged at 12,000 rpm for 15 min at 4 °C, and the supernatant was transferred to a new tube. Protein quantification and denaturation were performed according to the instructions of the BCA kit. The denatured supernatant containing 15 μg of protein was loaded into the gel and separated. Then, the gel was transferred to a PVDF membrane. The membrane was blocked with 5% skim milk at room temperature for 2 h, and then incubated overnight at 4 °C with primary antibodies (anti-claudin-1: 1:500, anti-claudin-7: 1:1000, anti-occludin: 1:2000) in 1 × TBST buffer. After removing the primary antibody solution and washing with 1 × TBST buffer, the membrane was incubated with HRP-labeled secondary antibody. Finally, the membrane was placed on a chemiluminescence imager for detection and imaging. The expression of glyceraldehyde-3-phosphate dehydrogenase (GAPDH) (1:5000) was used as an internal reference for claudin-1, claudin-7, and occludin.

### 2.12. LDF Measurement of Local Skin Blood Flow Values

The rats were placed in a prone position, and the LDF optical fiber probe (Moor VMS, Moor Instruments, Axminster, UK) was fixed on the local blood vessels of the rat’s ear. The local skin blood flow perfusion of the ear was recorded before and after administration, after the instrument reading was stable for 5 min. The basal blood flow values of each group of rats were continuously monitored for 30 min before administration, and the values were recorded. Then, the corresponding test solution was applied to the inner side of the rat’s ear in an equal area, and the blood flow perfusion was recorded every 10 min after administration, with the average value taken. Monitoring and observation were continued until 60 min. The detection site of the LDF optical fiber probe remained unchanged during the experiment. Moor V MS data analysis V2.0 software was used to analyze the average blood flow perfusion of the local skin of the rat’s ear before administration and at 10, 20, 30, 40, 50, and 60 min after administration, with the unit being the general unit PU of the instrument. The final results were expressed as the ratio of the blood flow value at each time point after administration to the basal blood flow value before administration.

### 2.13. Data Analysis

All experimental results were statistically analyzed using SPSS 26.0, Microsoft Excel, and Origin 2021. Normality (Shapiro–Wilk test) and homogeneity of variance (Levene’s test) were assessed prior to statistical testing. For single comparisons between two groups, standard unpaired two-tailed t-tests were used. For multiple comparisons, one-way analysis of variance (ANOVA) was performed, followed by Dunnett‘s post hoc test for pairwise comparisons between each treatment group and the control group. A *p*-value of less than 0.05 was considered statistically significant.

## 3. Results

### 3.1. GC-MS/MS

The volatile oil components of *Evodia rutaecarpa* were identified via gas chromatography–mass spectrometry. The results showed that the volatile oil contained 33 components, with the main components being β-pinene, γ-terpinene, (Z)-β-ocimene, terpinyl acetate, and 3-carene, with contents of 42.35%, 25.71%, 11.59%, 3.22%, and 2.96%, respectively. Therefore, the top three components in terms of content were selected for molecular docking and molecular dynamics simulation studies.

### 3.2. Biosafety Assessment

#### 3.2.1. Cytotoxicity Evaluation

**Figure 1:**
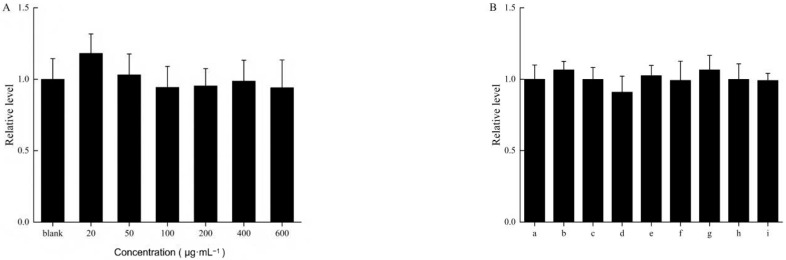
(**A**) Evaluation results of cytotoxicity of VO-ER cells at different concentrations. (**B**) Evaluation results of cytotoxicity before and after the combination of different concentrations of VO-ER and TGP. (a is the blank group, b is the negative control group, c is the low-dose group of VO-ER, d is the medium-dose group of VO-ER, e is the high-dose group of VO-ER, f is the TGP group, g is the low-dose VO-ER+TGP group, h is the medium-dose VO-ER+TGP group, and i is the high-dose VO-ER+TGP group).

All experimental results were statistically analyzed in accordance with the requirements of the Section 2.13. As shown in Figure 1, there were no significant differences in cell survival rates among the VO-ER concentration groups, the TGP group, and its combination group compared to the control group (*p* > 0.05), indicating that under the experimental conditions of this study, none of the administration groups exhibited cytotoxicity to HaCaT cells.

#### 3.2.2. Skin Erythema Analysis

**Figure 2:**
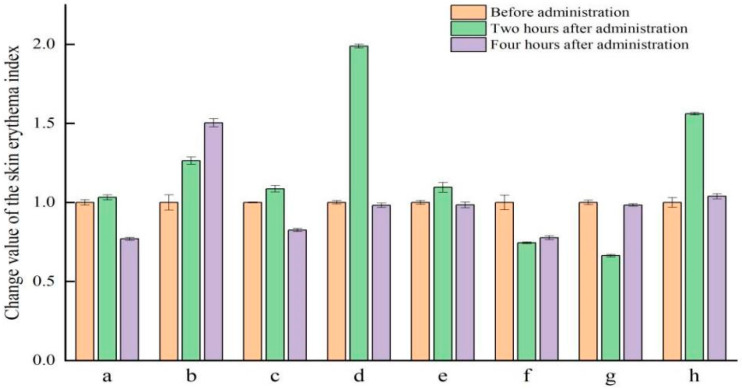
Changes in skin hemoglobin levels before and after administration in different groups (mean ± SD, *n* = 6). (a is blank, b is 1% VO-ER, c is 3% VO-ER, d is 5% VO-ER, e is TGP, f is 1% VO-ER+TGP, g is 3% VO-ER+TGP, and h is 5% VO-ER+TGP).

Skin irritation before and after the combined administration of VO-ER and TGP was evaluated through in vivo skin erythema analysis. As shown in Figure 2, compared to before administration, the hemoglobin levels in each group generally showed a trend of first increasing and then decreasing, indicating that VO-ER may cause some acute skin irritation, but it can quickly return to the initial state.

### 3.3. Ex Vivo Skin Permeation Study

**Figure 3:**
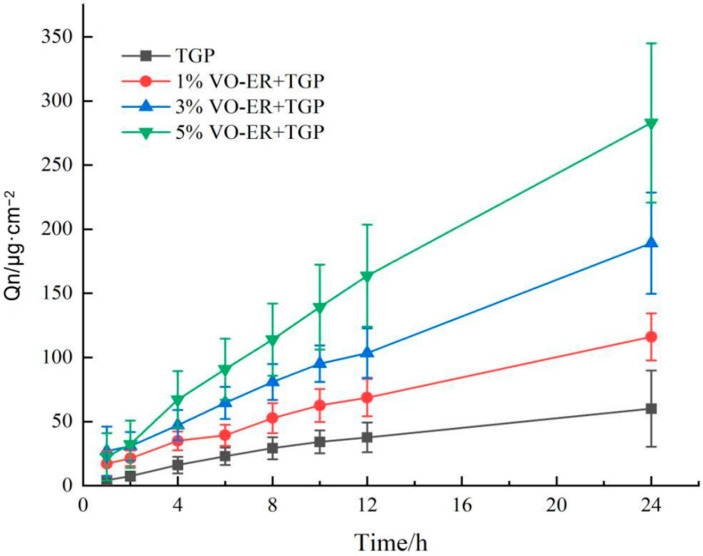
The permeability curves of PF in rat under the action of VO-ER (mean ± SD, *n* = 6).

The effect of VO-ER on the transdermal permeation of TGP was investigated through ex vivo skin permeation experiments, and the concentration of PF was determined using the established high-performance liquid chromatography method. The method had a good linear range of 0.11 μg·mL^−1^ to 220 μg·mL^−1^, with R^2^ = 0.9998. The results of the ex vivo skin permeation experiments are shown in Figure 3 and Table 1. As shown in Figure 3, compared to the TGP group, 3% and 5% VO-ER significantly enhanced the skin permeation of PF (*p* < 0.05). By contrast, although 1% VO-ER also promoted the transdermal absorption of PF to a certain extent, the effect was not statistically significant (*p* > 0.05). The QER values of 1%, 3%, and 5% VO-ER were 1.40, 2.32, and 3.83, respectively, indicating that VO-ER at different concentrations all enhanced the permeation of TGP, and the enhancement effect was ranked as follows: 5% VO-ER > 3% VO-ER > 1% VO-ER.

### 3.4. In Vivo Transdermal Absorption Behavior Study

**Figure 4:**
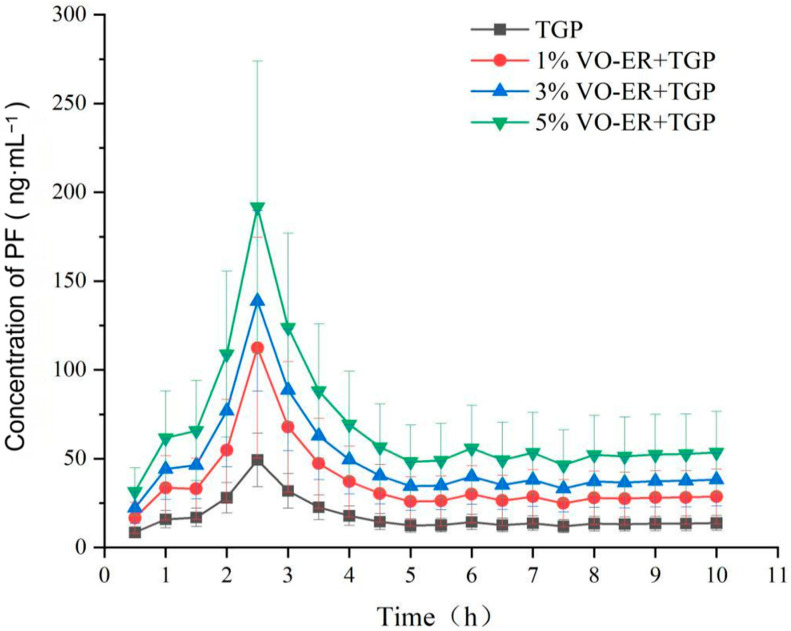
Subcutaneous drug concentration–time curves of PF before and after transdermal administration of the combination of VO-ER and TGP (mean ± SD, *n* = 4).

The permeability of TGP in vivo was significantly enhanced by different concentrations of VO-ER, as shown in Figure 4 and Table 2. VO-ER accelerated drug absorption and increased drug permeation, showing a concentration-dependent effect. From the parameters in Table 2, it can be seen that 5% VO-ER had the best penetration enhancement effect, followed by 3% and 1% VO-ER.

### 3.5. ATR-FTIR

**Figure 5:**
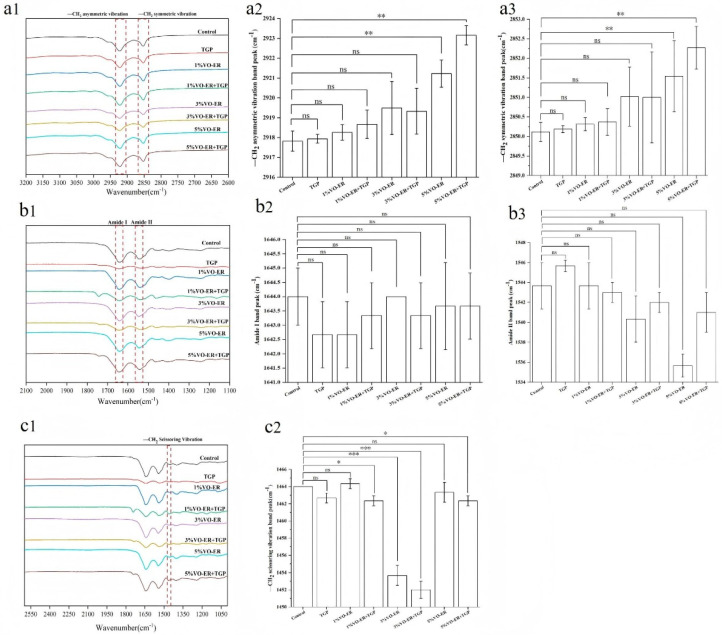
(**a1**–**a3**) Changes in the positions of C-H asymmetric and symmetric vibration peaks indicated by ATR-FTIR scanning. (**b1**–**b3**) Changes in the positions of amide Ι and amide ΙΙ band vibration peaks after ATR-FTIR scanning. (**c1**,**c2**) Changes in the position of the CH shear vibration peak indicated by ATR-FTIR scanning (mean ± SD, *n* = 3). ns *p* > 0.05, * *p* < 0.05, ** *p* < 0.01, *** *p* < 0.001.

The effects of VO-ER and TGP on intercellular lipids in the SC layer were investigated via ATR-FTIR. Among them, the asymmetric (νasCH_2_, 2920 cm^−1^) and symmetric (νsCH2, 2850 cm^−1^) stretching vibrations of -CH_2_ were used to characterize the conformation of intercellular lipid chains [15]. The results are shown in Figure 5a1–a3. Compared to the control group, except for the TGP group, the wavenumbers of the asymmetric vibration absorption peak of -CH_2_ and the symmetrical vibration absorption peak of -CH_2_ in the skin lipids of each administration group all underwent a certain degree of blue shift, indicating that the lipids in the treatment group underwent a certain degree of change. Moreover, a higher concentration of VO-ER induced a greater blue shift, indicating that different concentrations of VO-ER all had an impact on skin lipids, showing a concentration dependence.

In addition, ATR-FTIR was utilized to detect changes in the skin keratin structure. As shown in Figure 5b1–b3, the dashed boxes represent the molecular vibrational absorption peaks (amide I and amide II bands) of keratin in the SC. The FTIR spectra of the skin from each treatment group showed different peaks, with signals related to keratin typically located near 1650 cm^−1^ (amide I band) and 1550 cm^−1^ (amide II band). Compared to the control group, all administration groups showed certain shifts (left or right shift) in the vibrations of the amide I and amide II bands. The VO-ER group exhibited a more pronounced shift in the amide II band. These findings indicated that the keratin structure in the treated groups underwent certain changes, becoming more disordered. Due to the interaction between VO-ER and the stratum corneum, conformational changes were induced, leading to keratin denaturation. This promoted the loosening of the skin structure, thereby enhancing the transdermal absorption of PF.

The shear vibration of -CH_2_ groups in the FTIR spectra also received attention. The “width” of its infrared absorption peak generally refers to the full width at half maximum (FWHM), referring to the width of the peak at half of its maximum height. The FWHM of the -CH_2_ shear vibration reflects the content of the orthorhombic lattice in the skin, which is inversely proportional to the skin’s barrier function. Compared to the control group (Figure 5c1–c2), all treatment groups showed an increase in FWHM, and this increase was concentration-dependent. These results indicated a reduction in the skin’s barrier function, which is more conducive to promoting transdermal drug penetration.

### 3.6. Molecular Docking

It was found that no hydrogen bonds were formed in any of the component–target combinations. The binding forces between the components and targets mainly relied on non-polar interactions, with “hydrophobic interactions/van der Waals forces” as the dominant types of binding forces. These forces are highly compatible with the “lipophilic microenvironment” of the skin stratum corneum. The results are shown in Figure S1. Ea can reflect the intensity of intermolecular interactions to a certain extent. A negative value indicates significant intermolecular interactions, and a stronger interaction is reflected by a more negative value. The calculated hydrogen bond energies of all systems are shown in Table 3. From the Ea values, it can be observed that the three components of VO-ER exhibit higher affinity for cholesterol and keratins than for ceramides and free fatty acids. These research results were mutually corroborated by the findings of the ATR-FTIR studies.

### 3.7. MD

**Figure 6:**
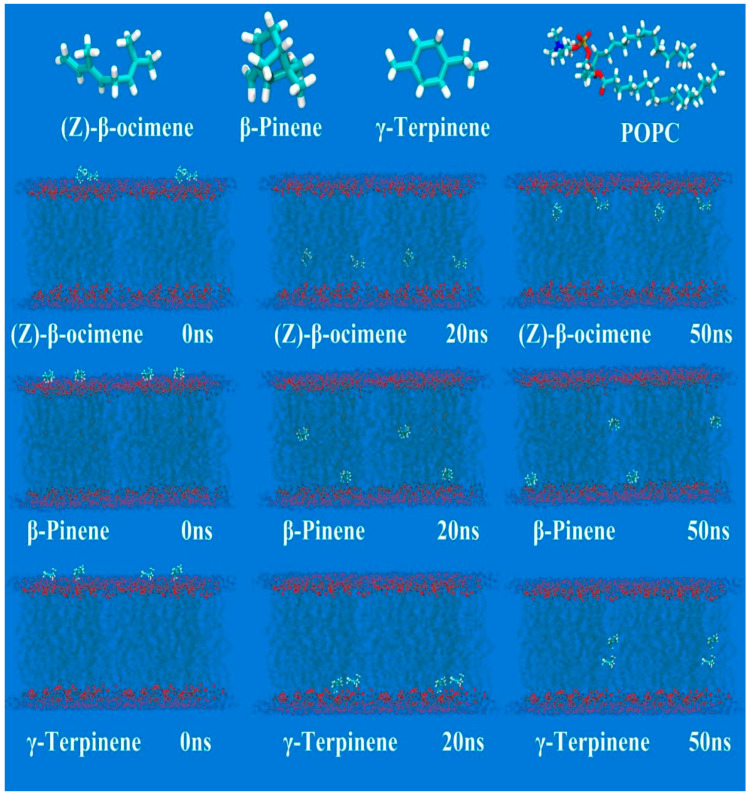
Movement trajectories of β-pinene, γ-terpinene, and (Z)-β-ocimene in the lipid bilayer.

**Figure 7:**
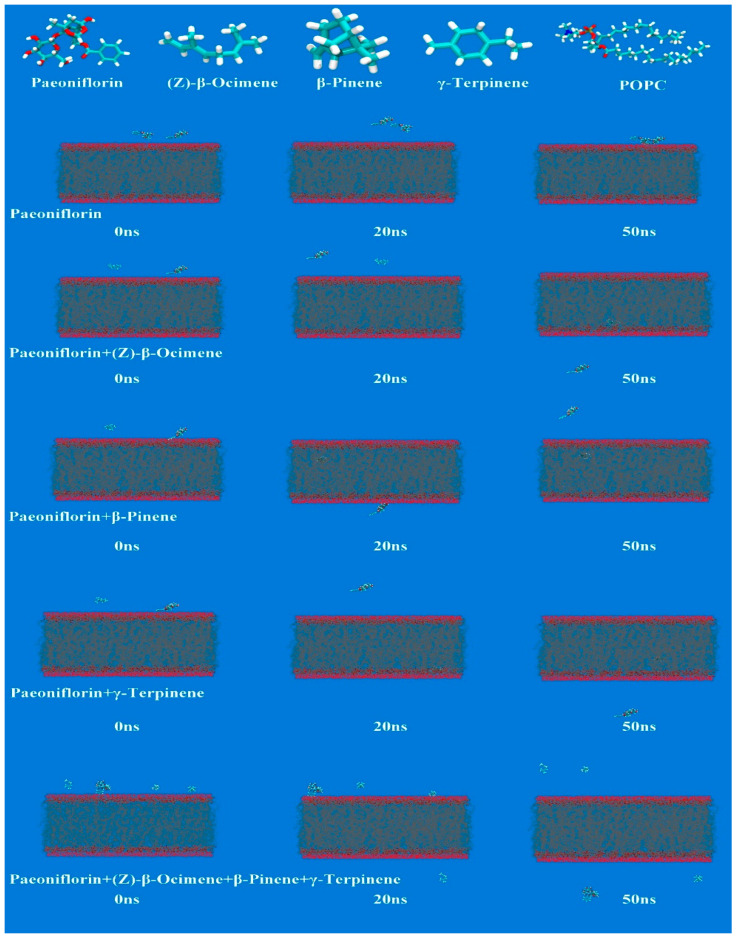
The movement trajectories of PF, β-pinene, γ-terpinene, and (Z)-β-ocimene in the lipid bilayer.

Through the curve calculation of mean shift (MSD) and simulation time, a 50 ns dynamic simulation process was generated, and the diffusion coefficients (D) of PF, AP, β-pinene, γ-terpinene, and(Z)-β-ocimene in the lipid bilayer of each group were obtained. The results are shown in Figure 6 and Figure 7. The results showed that within the lipid force field, β-pinene, γ-terpene, and (Z)-β-ocimene could all enter the phospholipid bilayer and bind to lipids within 50 ns. PF could not pass through the phospholipid bilayer within 50 ns, but it could pass through at 50 ns after being combined with β-pinene and (Z)-β-ocimene. This indicated that β-pinene and (Z)-β-ocimene can combine with lipid components, altering the structure of the phospholipid bilayer and allowing the hydrophilic component PF to pass more easily through the phospholipid bilayer. The MSD plots of the different terpenes and PF–terpene complexes are presented in Figure S2.

### 3.8. CLSM

**Figure 8:**
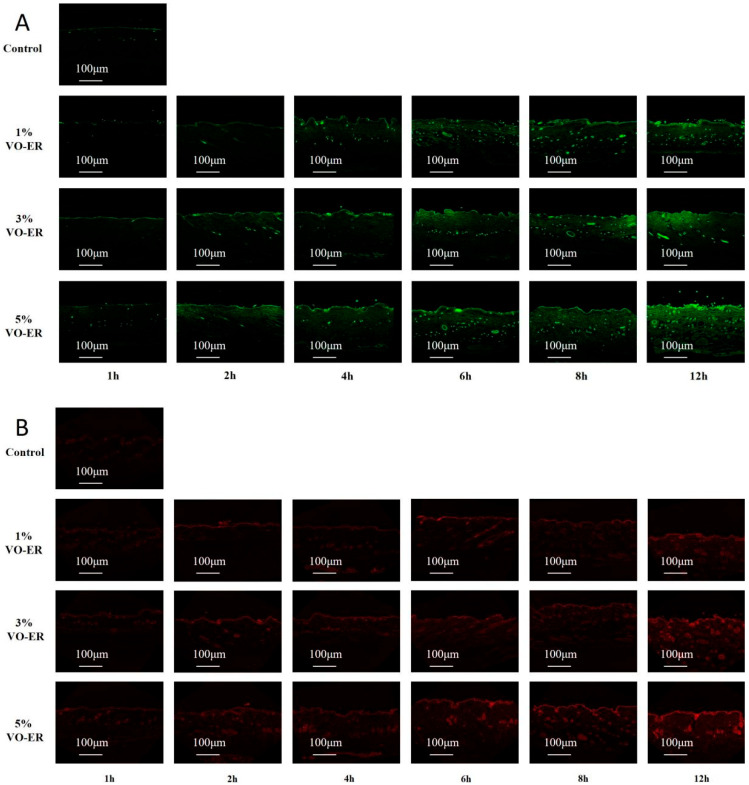
Graphs of the penetration amount and depth of fluorescent dyes at different time periods after administration of VO-ER at different concentrations. (**A**)—sodium fluorescein; (**B**)—Nile Red.

The experiment found that compared to the control group, the fluorescence intensity, penetration amount, and penetration depth of the two fluorescent dyes, sodium fluorescein and Nile Red, all increased to varying degrees after being administered different concentrations of VO-ER. Moreover, a higher concentration of VO-ER induced a more obvious effect. By comparing the fluorescence intensity, penetration volume, and penetration depth of fluorescent dyes at different administration times with the same concentration of VO-ER, it was found that the fluorescence intensity, penetration volume, and penetration depth all increased with an increase in administration time, which was consistent with the results of the ex vivo transdermal absorption studies, indicating that VO-ER may improve skin permeability. An increase in the skin penetration depth of the drug promoted the transdermal absorption of TGP. The results are shown in Figure 8 (A is sodium fluorescein and B is Nile Red).

### 3.9. Western Blot

**Figure 9:**
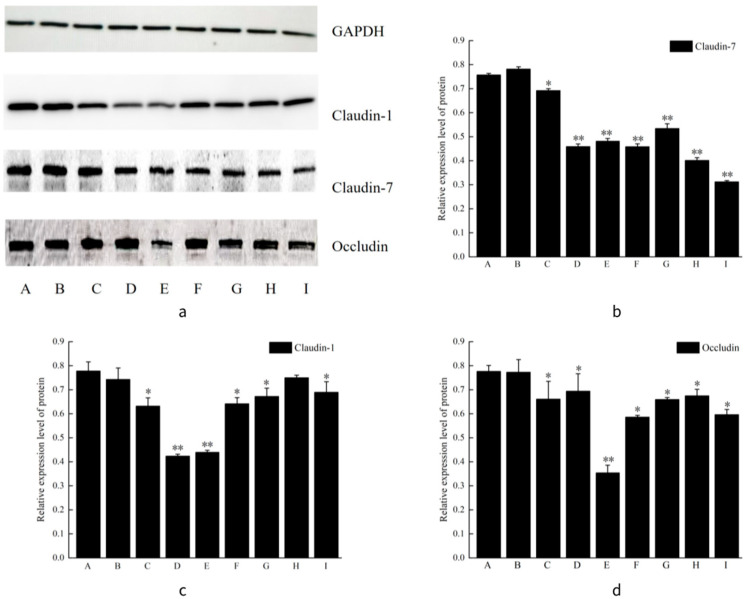
Effect of VO-ER combined with TGP on the expression of tight junction proteins before and after the combination of VO-ER and TGP (**a**). Relative expression levels of tight junction proteins before and after the combination of VO-ER and TGP (**b**–**d**). (A is the blank group, B is the negative control group, C is the low-dose group of VO-ER, D is the medium-dose group of VO-ER, E is the high-dose group of VO-ER, F is the TGP group, G is the low-dose VO-ER+TGP group, H is the medium- dose VO-ER+ TGP, and I is the high-dose VO-ER+TGP group). Compared with the control group, * *p* < 0.05, ** *p* < 0.01.

The effects of different concentrations of VO-ER combined with TGP on tight junction proteins in the skin were explored. The experimental results are shown in Figure 9. As shown in Figure 9b–d, compared to the blank group, the protein expression levels of claudin-1, claudin-7, and occludin were significantly decreased (*p* < 0.05) in all dose groups of VO-ER, the TGP group, and the combination groups, exhibiting a concentration-dependent manner.

In contrast to the VO-ER alone groups, the expression of claudin-1 and occludin was increased in the corresponding combination groups, whereas the expression of claudin-7 was decreased. This indicated that TGP may attenuate the inhibitory effects of VO-ER on claudin-1 and occludin but enhance the inhibitory effect on claudin-7. In conclusion, VO-ER significantly reduced the expression of tight junction proteins in the skin, including claudin-1, claudin-7, and occludin (*p* < 0.05), thereby loosening tight junctions in the skin.

### 3.10. LDF Was Used to Measure the Local Bloodflow Value of the Skin

**Figure 10:**
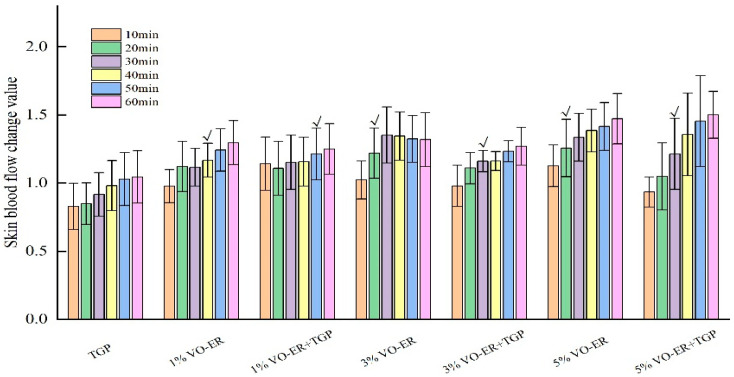
Comparison of changes in blood perfusion volume at different time points among different administration groups (mean ± SD, *n* = 6). Compared to before administration, blood flow changes began to show differences between groups after administration, which are indicated by √.

**Figure 11:**
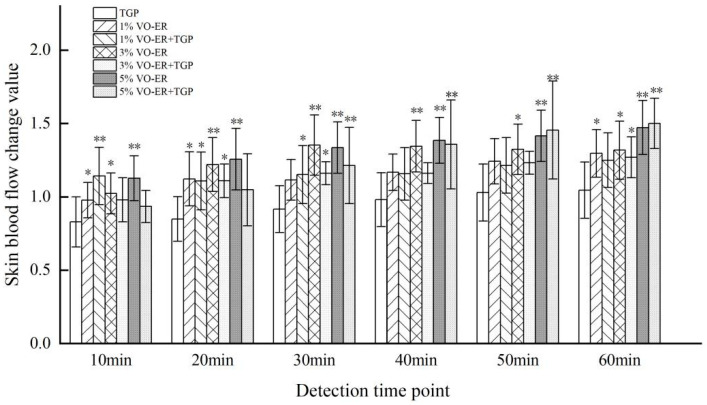
Comparison of changes in blood perfusion volume among different administration groups at the same detection time point (mean ± SD, *n* = 6). Compared to the TGP group at the same detection time point, * *p* < 0.05, ** *p* < 0.01.

The effects of different concentrations of VO-ER combined with TGP on local skin blood flow were explored. The experimental results are shown in Figure 10 and Figure 11. As can be seen in Figure 10, after applying 1% VO-ER, 3%VO-ER, and 5% VO-ER, skin blood flow was significantly accelerated at 40 min, 20 min, and 20 min, respectively, indicating that a higher concentration of VO-ER increased blood flow. Compared to the application of volatile oil alone, the time for different concentrations of VO-ER to significantly accelerate skin blood flow after being combined with TGP was prolonged. Overall, the blood flow values at each time period after VO-ER was combined with TGP were slightly lower than those when using the volatile oil alone, indicating that TGP may slow down the local blood flow velocity in the ears of rats.

As can be seen in Figure 11, after applying the liquid medicine, the change values of skin blood flow in each group were the largest at 60 min after administration. Among them, the group with 5% VO-ER combined with TGP had the largest change value, followed by the 5% VO-ER group, 3% VO-ER group, 1% VO-ER group, 3% VO-ER combined with TGP group, and 1% VO-ER combined with TGP group. This indicated that VO-ER can significantly change the blood flow of the skin, and it showed a concentration dependence.

## 4. Discussion

Transdermal drug delivery has emerged as a pivotal non-invasive strategy in pharmaceutical research and clinical practice, with the stratum corneum (SC) being widely recognized as the primary barrier to effective drug penetration. Chemical penetration enhancers (PEs) are commonly employed to overcome this barrier [16], yet their application is often limited by insufficient efficacy at low concentrations or skin irritation at high doses [17]. By contrast, essential oils derived from traditional Chinese medicines (TCMs) have garnered increasing attention owing to their favorable safety profiles and potential penetration-enhancing properties [18]. *Evodia rutaecarpa*, a classic TCM with a long history of topical use, has been documented in ancient texts for its “opening couli” effect, and its volatile oil (VO-ER) is a key active component. While previous studies have confirmed that VO-ER can promote the transdermal absorption of PF—the main bioactive constituent of TGP—the underlying mechanism remains poorly understood. Addressing this gap, the present study systematically investigated the enhancement effect of VO-ER on TGP transdermal delivery from the perspective of “skin physical barrier–tight junction proteins–cutaneous microcirculation,“ thereby providing a scientific interpretation of VO-ER’s traditional “opening couli” function.

A critical finding of this study is that VO-ER significantly enhanced the ex vivo and in vivo transdermal permeation of PF in a concentration-dependent manner, which was corroborated by both modified Franz diffusion cell experiments and microdialysis assays. Mechanistically, our multi-dimensional analyses revealed that VO-ER exerts its effects through synergistic regulation of the skin’s physical and molecular barriers, as well as cutaneous microcirculation. Regarding the skin’s physical barrier, ATR-FTIR spectroscopy demonstrated that VO-ER induces a blue shift in the asymmetric (νasCH_2_, 2920 cm^−1^) and symmetric (νsCH_2_, 2850 cm^−1^) stretching vibrations of -CH_2_ groups in SC lipids, accompanied by an increase in the full width at half maximum (FWHM) of the -CH_2_ scissoring vibration. These spectral changes indicated that VO-ER disrupts the ordered packing of intercellular lipids, increases lipid fluidity, and reduces the skin’s barrier function [19]—effects that were further validated by molecular docking and molecular dynamics (MD) simulations. Molecular docking results showed that the major components of VO-ER (β-pinene, γ-terpinene, and (Z)-β-ocimene) exhibit high affinity for skin lipids (especially cholesterol) and keratins (KRT1, KRT10, and KRT14), primarily through hydrophobic interactions and van der Waals forces. MD simulations further confirmed that these terpenoid components can insert into the phospholipid bilayer, alter its structure, and enable the hydrophilic PF to traverse the bilayer—an event that did not occur when PF was administered alone. Confocal laser scanning microscopy (CLSM) observations using fluorescent probes (sodium fluorescein and Nile Red) further supported these findings, showing that VO-ER increases the penetration depth and amount of drugs in the skin, which is consistent with the enhanced permeability of the SC.

Epidermal tight junctions (TJs) are widely recognized as the “primary regulators” governing the epidermal permeability barrier [20]. As the core components of skin TJ structures, TJ proteins can be classified into transmembrane proteins and cytoplasmic proteins. Notably, claudins and occludin are key transmembrane constituents of TJs. Reduced expression of these proteins weakens adhesion between epidermal keratinocytes, subsequently impairing the skin’s physical barrier function [21]. For instance, claudin-1 knockout mice die shortly after birth due to severe skin barrier dysfunction and excessive transepidermal water loss [22,23]. Beyond its role as a structural TJ protein, occludin also regulates the paracellular diffusion of hydrophilic substances in the skin—a function closely associated with skin hydration.

Cytoplasmic TJ proteins primarily include the zonula occludens (ZO) protein family and cingulin. Their primary function is to anchor transmembrane TJ proteins to the cytoskeleton, thereby facilitating the formation of an integrated TJ network [24]. Given that alterations in TJ protein expression directly disrupt the skin’s barrier function, we conducted a study using HaCaT cells to investigate the effects of VO-ER alone and in combination with TGP on TJ protein expression. Western blot analysis revealed that VO-ER concentration-dependently downregulated the expression of claudin-1, claudin-7, and occludin in HaCaT cells. Claudins and occludin are critical transmembrane proteins that maintain the integrity of epidermal TJs. Reduced expression of these proteins weakens the adhesive force between keratinocytes, thereby loosening the skin’s barrier function and facilitating paracellular drug transport. Notably, when combined with TGP, the downregulatory effect of VO-ER on claudin-1 and occludin was attenuated, while its effect on claudin-7 was enhanced. This observation suggested a potential interactive effect between TGP and VO-ER on TJ protein expression, which may reflect the “compatibility” principle of TCMs—a phenomenon that has not been reported in previous studies on VO-ER.

In addition to barrier modulation, VO-ER also improves cutaneous microcirculation, which plays an indirect but important role in transdermal drug delivery. Laser Doppler flowmetry (LDF) measurements showed that VO-ER increased local skin blood flow in a concentration-dependent manner, accelerating the elimination of PF from the epidermis to dermal capillaries and establishing a “sink effect” that drives sustained drug penetration. This finding aligns with the TCM property of *Evodia rutaecarpa* (pungent and hot, capable of promoting blood circulation) and is supported by modern pharmacological studies indicating that *Evodia rutaecarpa* extracts have vasodilatory effects. Interestingly, administration of TGP alone reduced skin blood flow, while when TGP was combined with VO-ER, it partially alleviated the increase in skin blood flow caused by VO-ER and the downregulation of tight junction proteins (claudin-1 and occludin). In the combined group, PF showed a “buffering” effect on the increase in blood flow caused by VO-ER, suggesting that PF’s regulation of microvascular function may interact with the vasodilatory effect of VO-ER. This bidirectional regulation may reflect the compatible mode of “harmony of cold and heat” in traditional Chinese medicine—the “acidic and cold” nature of white peony inhibits the “pungent and hot” nature of *Evodia rutaecarpa* to a certain extent. In the rat model of bile stasis [25], after PF intervention, the mRNA and protein levels of ZO-1, occludin, and claudin-3 in liver tissue significantly increased, indicating that PF has a protective effect on the integrity of tight junctions, which is consistent with the observations of this study. At the molecular level, we speculate that PF and the active components in VO-ER may competitively bind to certain common targets, such as PKC and TRPV1 [26], because these two signaling pathways are involved in regulating microcirculation function and the expression of tight junction proteins. The specific mechanisms of these interactions need further in-depth study.

Safety is a critical consideration for penetration enhancers [27], and the present results confirmed that VO-ER exhibits minimal skin irritation. The cytotoxicity evaluation results showed that in the HaCaT cell line, VO-ER, TGP, and their combination used in the experiment did not exhibit any cytotoxicity. In vivo erythema analysis showed that the transient increase in skin hemoglobin levels induced by VO-ER returned to baseline within 4 h. This safety profile is particularly advantageous compared to synthetic chemical enhancers, making VO-ER a promising candidate for clinical application.

It should be noted that although this study has established the mechanistic evidence that VO-ER promotes the transdermal absorption of PF in healthy skin, it remains to be clarified whether this permeation-promoting effect can translate into better anti-inflammatory efficacy in diseased skin. Future research is necessary to introduce disease models in order to further bridge the gap between mechanism exploration and clinical application.

The innovations of this study are threefold: First, it is the first study to systematically elucidate the multi-dimensional mechanism of VO-ER in enhancing TGP transdermal delivery, integrating analyses of lipid barrier structure, TJ protein expression, and microcirculation. Most volatile oils and terpenoid penetration enhancers mainly act on the intercellular lipids or keratin structures. However, VO-ER further regulates the expression of tight junction proteins claudin/occludin and local blood flow. That is, through the multi-mechanism of “lipid barrier + tight junction proteins + microcirculation,” it achieves a better penetration effect and has good safety. This approach goes beyond previous studies focusing solely on lipid modulation. Second, it provides a direct link between the traditional “opening couli” effect of *Evodia rutaecarpa* and modern molecular/cellular mechanisms, thereby bridging TCM theory and contemporary pharmacology. Third, it uncovers an interactive effect between VO-ER and TGP in skin physiology, which contributes to a deeper understanding of TCM compatibility in transdermal drug delivery.

## 5. Conclusions

In summary, this study demonstrates that VO-ER enhances TGP transdermal absorption through three interconnected mechanisms: disrupting the SC lipid barrier, downregulating TJ protein expression, and improving cutaneous microcirculation. These findings not only scientifically validate the traditional “opening couli” function of *Evodia rutaecarpa* but also provide a theoretical basis for VO-ER as a safe and effective natural penetration enhancer. Furthermore, this work offers a paradigm for investigating TCM-derived penetration enhancers and supports the development of novel topical formulations containing *Evodia rutaecarpa* and *Paeonia lactiflora* for the treatment of immune-related or inflammatory skin diseases.

## Figures and Tables

**Table 1:** Parameters for in vitro skin permeation kinetics of PF before and after the combination of VO-ER with TGP (mean ± SD, *n* = 6).

Group	Q24 h (μg·cm^−2^)	Jss (μg·cm^−2^·h^−1^)	QER
TGP	60.04 ± 29.74	3.20	1.00
1% VO-ER + TGP	116.06 ± 18.35 *	4.49	1.40
3% VO-ER + TGP	189.10 ± 39.42 **	7.43 *	2.32
5% VO-ER+TGP	282.89 ± 62.13 **	12.25 **	3.83

Note: Compared to TGP group, * *p* < 0.05 indicates a significant difference, ** *p* < 0.01 indicates an extremely significant difference.

**Table 2:** Main pharmacokinetic parameters of PF in rats after transdermal administration of the combination of VO-ER with TGP (mean ± SD, *n* = 4).

	Group	TGP	1% VO-ER+TGP	3% VO-ER+TGP	5% VO-ER+TGP
Parameters					
C_max_ (ng·mL^−1^)		49.27 ± 15.08	112.41 ± 62.19	138.77 ± 50.86 *	191.69 ± 82.20 *
T_max_ (h)		2.50 ± 0.00	2.50 ± 0.00	2.50 ± 0.00	2.50 ± 0.00
T_1/2_ (h)		116.37 ± 0.00	116.37 ± 0.00	116.37 ± 0.00	116.37 ± 0.00
AUC_0–10 h_ (ng·h·mL^−1^)		168.03 ± 51.45	352.12 ± 189.21 *	466.72 ± 182.56 *	653.66 ± 280.29 *
AUC _(1–∞)_ (ng·h·mL^−1^)		2444.52 ± 748.48	5101.50 ± 2737.01 *	6785.46 ± 2662.24 *	9509.29 ± 4077.68 *

Note: Compared to TGP group, * *p* < 0.05.

**Table 3:** Binding energies of β-pinene, γ-terpinene, and (Z)-β-ocimene with skin components.

Components	Binding Energy (kcal/mol)		
	β-Pinene	γ-Terpinene	(Z)-β-Ocimene
Ceramide 1	−2.1	−2.7	−2.6
Ceramide 3	−2.5	−2.9	−2.6
Ceramide 6	−2.0	−2.6	−2.6
Ceramide 7	−2.0	−2.4	−2.4
Tetracosanoic acid	−1.8	−2.6	−2.5
Hexacosanoic acid	−1.7	−2.4	−2.3
Cholesterol	−3.7	−4.7	−4.4
Keratin 1	−4.2	−3.43	−3.1
Keratin 10	−4.7	−4.5	−4.2
Keratin 14	−4.3	−3.8	−4.2

## Data Availability

The data presented in this paper can be made available upon request to the corresponding author.

## Supplementary Materials

The following supporting information can be downloaded at: https://www.mdpi.com/article/10.3390/pharmaceutics18040433/s1, Figure S1a: Docking diagram of β-Pinene, γ-Terpinene and (Z)-β-Ocimene with lipid components in the skin lipid layer. Figure S1b: Docking diagram of β-Pinene, γ-Terpinene and (Z)-β-Ocimene with keratins; Figure S2: Mean square displacement (MSD) plots of terpenes from Evodia rutaecarpa volatile oil and paeoniflorin (PF)-terpene complexes in the lipid bilayer. (A) Individual motion trajectories of the terpene components ( β-Pinene, γ-Terpinene and (Z)-β-Ocimene ) in the lipid bilayer; (B) Paeoniflorin (PF) alone; (C) PF in combination with β-Pinene; (D) PF in combination with γ-Terpinene; (E) PF in combination with (Z)-β-Ocimene; (F) PF in combination with all three terpene components (β-Pinene, γ-Terpinene and (Z)-β-Ocimene ). The MSD values are plotted as a function of simulation time. The slopes of the MSD curves correspond to the diffusion coefficients of the respective molecules or complexes.
