# Supplementary material for: Investigation of the Enhancement Effect of Evodia rutaecarpa Volatile Oil on Transdermal Delivery of Total Glucosides of Paeony: Mechanistic Insight Based on Interactions Among Drug, Enhancer, and Skin
Source: Pharmaceutics. 2026 Mar 31;18(4):433. doi: 10.3390/pharmaceutics18040433 (PMC13119413; doi:10.3390/pharmaceutics18040433)
Supplement: Supplementary file 1 [file pharmaceutics-18-00433-s001.zip › pharmaceutics-4162513-supplementary.pdf]

pharmaceutics

MDPI

Supplementary Materials

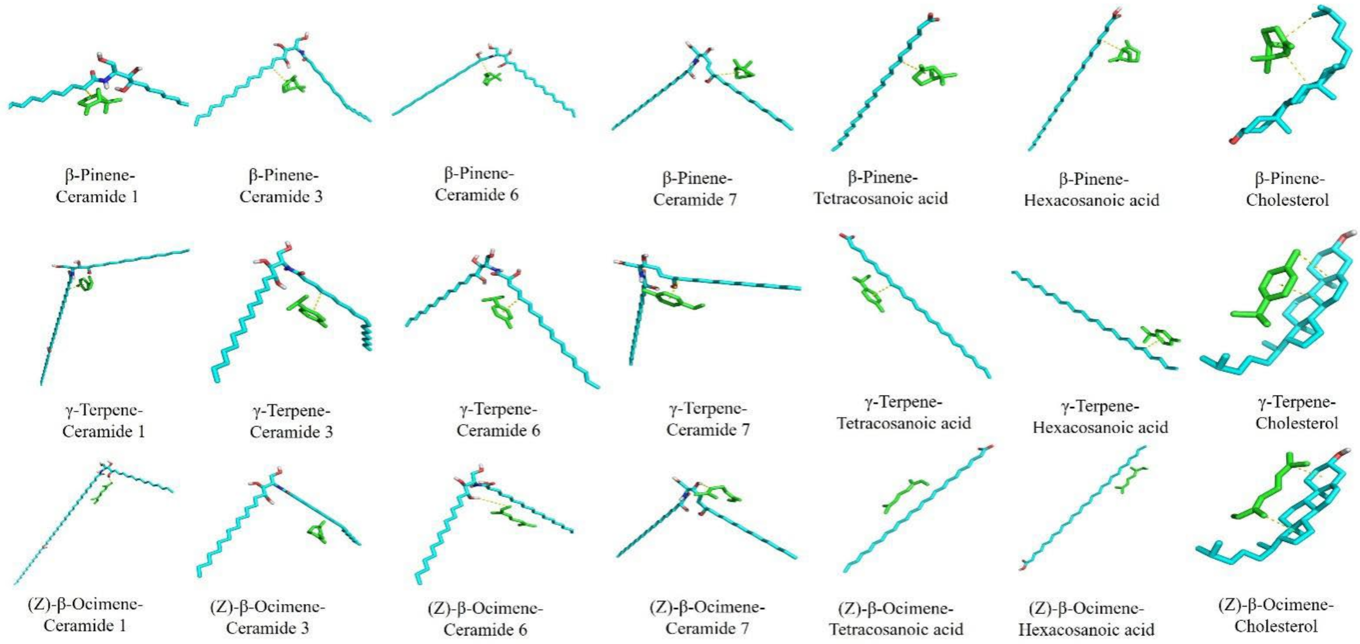

Figure S1a: Docking diagram of β-Pinene, γ-Terpinene and (Z)-β -Ocimene with lipid components in the skin lipid layer.

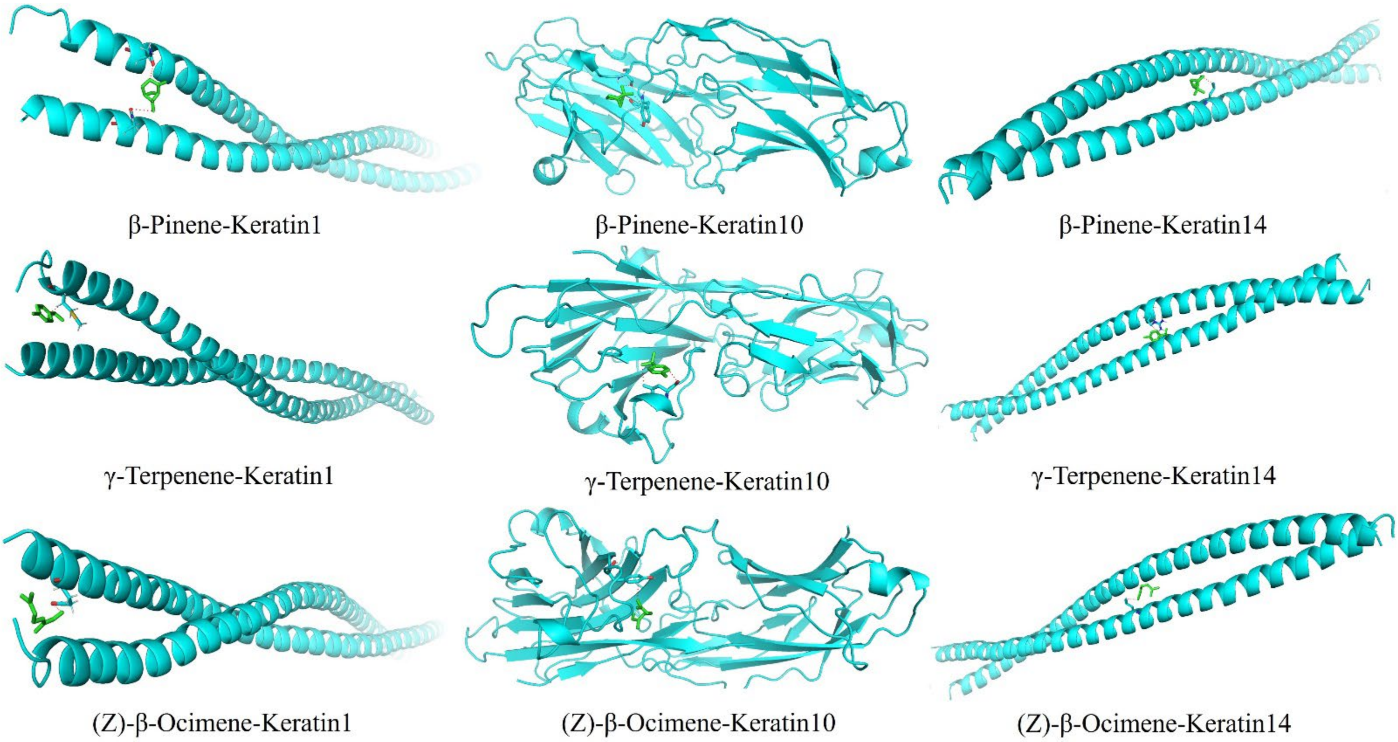

Figure S1b: Docking diagram of β-Pinene, γ-Terpinene and (Z)-β -Ocimene with keratins.

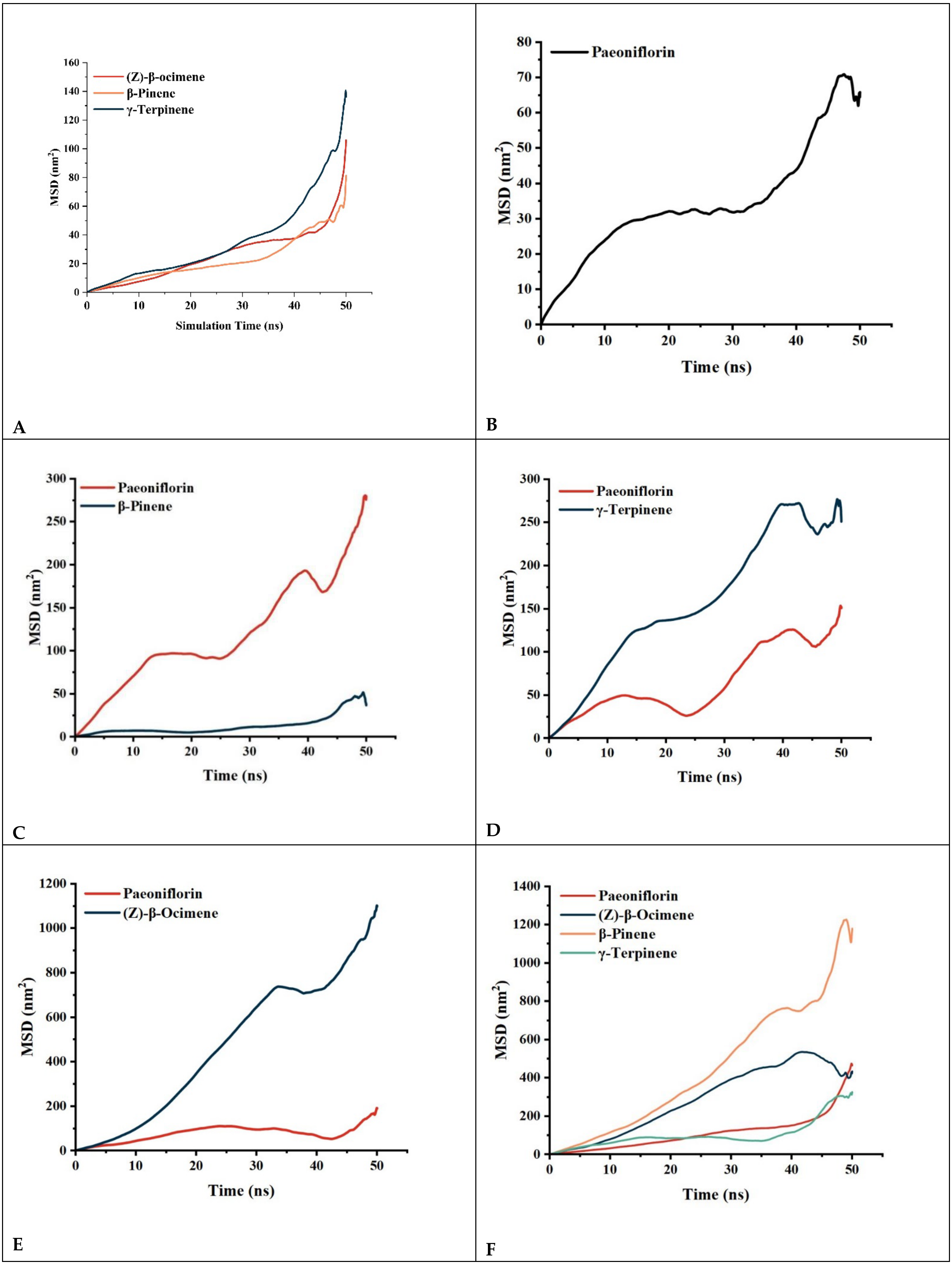
(Z)-β-ocimene
β-Pinene
γ-Terpinene
MSD (nm2)
Simulation Time (ns)
A
Paeoniflorin
MSD (nm2)
Time (ns)
B
Paeoniflorin
β-Pinene
MSD (nm2)
Time (ns)
C
Paeoniflorin
γ-Terpinene
MSD (nm2)
Time (ns)
D
Paeoniflorin
(Z)-β-Ocimene
MSD (nm2)
Time (ns)
E
Paeoniflorin
(Z)-β-Ocimene
β-Pinene
γ-Terpinene
MSD (nm2)
Time (ns)
F

Figure S2: Mean square displacement (MSD) plots of terpenes from *Evodia rutaecarpa* volatile oil and paeoniflorin (PF)-terpene complexes in the lipid bilayer. (A) Individual motion trajectories of the terpene components (β-Pinene, γ-Terpinene and (Z)-β -Ocimene) in the lipid bilayer; (B) Paeoniflorin (PF) alone; (C) PF in combination with β-Pinene; (D) PF in combination with γ-Terpinene; (E) PF in combination with (Z)-β -Ocimene; (F) PF in combination with all three terpene components (β-Pinene, γ-Terpinene and (Z)-β -Ocimene). The MSD values are plotted as a function of simulation time. The slopes of the MSD curves correspond to the diffusion coefficients of the respective molecules or complexes.
